# Empirical Use of Dopaminergic Agents for Restless Legs Syndrome Could be Associated With a Risk of Iatrogenic Parkinson’s Disease

**DOI:** 10.4021/jocmr1583w

**Published:** 2013-10-12

**Authors:** Yasuyuki Sugit

**Affiliations:** ugita Dermatology Clinic, 893-10 Nishiya-cho, Hodogaya-ku, Yokohama 240-0052, Japan. Email: sugiderm@yy.catv-yokohama.ne.jp

## To the Editor

Several drugs acting on the central nervous system (CNS) have been indicated for the treatment of restless legs syndrome (RLS), particularly pramipexol, which is a dopamine D2 receptor agonist [1, 2]. However, there is no significant evidence for a decrease of dopamine derivatives in patients with RLS, and association with Parkinson’s disease is not common. In fact, the use of dopaminergic agents for RLS has been merely empirical up to now. It can be easily envisaged that a continuous dopamine load in normal persons might lead to depletion of intrinsic dopamine production. Because the prevalence of RLS is surprisingly high, up to 10% of the general population might be considered candidates for dopaminergic therapy.

The original report of RLS by Ekbom in 1945 emphasized the usefulness of vasodilative agents [3, 4]. On the other hand, a dopamine D2 receptor agonist has been clearly mentioned as a strong vasodilator even in a widely used textbook of pharmacology [5]. Other CNS drugs recommended for RLS, such as benzodiazepines and opioids, also have vasodilative effects on the peripheral circulation in addition to effects on the CNS.

Recently, I have been proposed a new hypothesis suggesting that stimulation of the autonomic nervous system distributed to vessels in the lower legs could be the origin of the unpleasant sensations in RLS [6, 7]. Fifteen hours of exposure to gravity during the daytime, compressing the vessels in the lower legs, is required for occurrence of the unpleasant sensations. An abrupt change of blood flow in the legs due to lying down at night may cause abnormal tension of blood vessels, inducing unexpected excitement of the autonomic nervous system, especially in individuals with impaired vessel elasticity and distensibility. This provides a quite reasonable explanation for why the sensations occur upon lying down and disappear when walking. Circadian rhythm seems unlikely to account for it.

As well as CNS drugs, several drugs that act potent vasodilators would be worth trying for treatment of RLS. For example, it is often experienced that even vitamins E and B12 have shown some effectiveness for dermatology patients complaining night itching in the feet. Because of its primary action on the CNS, the use of dopamine D2 receptor agonist might be limited for severe cases that are deeply affecting the quality of life. In order to prevent an iatrogenic Parkinson’s disease, long-term use should be limited for patients who have associated Parkinson’s disease and it must be worth trying the combined use of a daily dose of 300 mg oral tocopherol acetate (vitamin E) which is supporting the peripheral blood circulation, and a daily dose of 750 μg oral mecobalamin (vitamin B12) which is preventing excessive excitement of the autonomic nerve system.
